# A Future of Smarter Digital Health Empowered by Generative Pretrained Transformer

**DOI:** 10.2196/49963

**Published:** 2023-09-26

**Authors:** Hongyu Miao, Chengdong Li, Jing Wang

**Affiliations:** 1 College of Nursing Florida State University Tallahassee, FL United States

**Keywords:** generative pretrained model, artificial intelligence, digital health, generative pretrained transformer, ChatGPT, precision medicine, AI, privacy, ethics

## Abstract

Generative pretrained transformer (GPT) tools have been thriving, as ignited by the remarkable success of OpenAI’s recent chatbot product. GPT technology offers countless opportunities to significantly improve or renovate current health care research and practice paradigms, especially digital health interventions and digital health–enabled clinical care, and a future of smarter digital health can thus be expected. In particular, GPT technology can be incorporated through various digital health platforms in homes and hospitals embedded with numerous sensors, wearables, and remote monitoring devices. In this viewpoint paper, we highlight recent research progress that depicts the future picture of a smarter digital health ecosystem through GPT-facilitated centralized communications, automated analytics, personalized health care, and instant decision-making.

## Introduction

As a very successful commercial product in the market, the big story of ChatGPT started with an adventure of a group of Google researchers in 2017 [1], who introduced the transformer structure based on an attention mechanism for a specific natural language processing (NLP) task—neural machine translation. OpenAI built ChatGPT upon this technology, and it is one of the fastest growing consumer applications [2]. The magic here is quantitative change leading to qualitative change: ChatGPT has significantly more model parameters than the original attention model. More specifically, Generative Pretrained Transformer–3.5 (GPT-3.5) [3] launched in November 2022 has 175 billion parameters and GPT-4.0 [4] released in March 2023 has 1 trillion parameters. For this reason, ChatGPT and its competitors including Google Bard and Facebook LLaMA are called large language models (LLMs). However, training LLMs is expensive, and the estimated cost falls between US $2 million and US $12 million nowadays [5]. OpenAI thus adopts a traditional business model for its groundbreaking product to “share” such costs primarily via monthly user subscription. In March 2023, ChatGPT alone reached 1.16 billion registered users [6]. Note that many users are attracted to LLMs because of the great potential of GPT tools in improving and renovating health care research and practice [7], including the current transformation happening in digitizing health care [8].

Digital health is a blooming field in which digital technology, medicine, behavior, health care, and community living are integrated for health improvement of both the population and individuals [9-15]. In particular, the development and deployment of innovative hardware and software (eg, wearable and implantable sensors and mobile apps) and communication technologies (eg, real-time augmented reality and streaming data platforms) lay the foundation of modern digital health. Within the past decade, numerous novel concepts and ideas in digital health have been generated to renovate our health care systems, triumphing over a variety of challenges in improving treatment efficacy, reshaping health care delivery, personalizing medical and behavioral intervention, etc. One representative example of digital medicine is deep brain stimulation for, for example, drug-resistant epilepsy [16], where medications are not in the traditional form of chemical compounds but implantable chips with computational intelligence for seizure onset detection and disruption. We are also witnessing the thriving of numerous mobile medical and health apps [17] for, for example, emergency medicine, chronic condition management, behavioral medicine, and mental health, all of which heavily rely on existing or novel communication technologies such as Bluetooth, 5G, Wi-Fi, and privacy protection instruments. The built-in complexity of digital health tools and their significantly large number of data elements generated through such tools naturally call for the customization and application of artificial intelligence (AI)–based devices and algorithms [18-20]. Therefore, it is not an exaggeration to say that we live in an era of intelligent digital health. Given the remarkable success of GPT in many fields, including but not limited to protein design [21], drug discovery [22], intelligent transportation [23], and health care research and practice [24], intelligent digital health is expected to become even smarter if appropriately integrated with LLMs such as ChatGPT.

In this viewpoint paper, we discuss how GPT tools could lead to smarter digital health, and we briefly describe certain areas of caution related to the use of GPT and similar AI tools in digital health education, research, and practice.

## Simplification and Streamlining of Communication to Reduce Clinician Burden

In digital health research and practice, communication has never been as simple as in-person conversations between patients and clinicians. Frequent communications among various hardware, software, backend cloud or streaming data platforms, engineers, data scientists, patients, and health care providers are the common scene for digital health applications. For instance, streaming time-series data are constantly measured by a variety of mobile sensors (eg, accelerometers, gyroscopes, thermometers, and photoplethysmography), and such data might be preprocessed and transferred in real time via secured Bluetooth, Wi-Fi, or 5G to centralized data cloud for storage, management, analysis, and mining by engineers, data scientists, or automated algorithms. The summarized results (eg, diagnostic or prognostic) may then be sent to health care providers for interpretation and decision-making, which are eventually conveyed to patients via, for example, mobile apps. The interesting thing is that despite the central roles of mobile sensors in such communication cycles, users currently do not have the option to directly “talk” to such sensors. It is not difficult to imagine how significantly the aforementioned communication cycle can be simplified if wearable or mobile sensors can passively or actively vocalize the health status (eg, symptoms, anomalies, and behavioral changes) they detect, and even give instant suggestions or make decisions such as emergent calls directly for the users. Technically, achieving this goal requires a better integrated electronic system, a higher marginal computing power, and a GPT-powered brain. If one may question the feasibility of such smarter sensor-based communication in digital health by pointing out that speakers and microphones are too bulky to fit in small chips, one must consider that Massachusetts Institute of Technology researchers have recently developed a paper-thin loudspeaker that is potentially wearable [25]. Note that many other industries are sharing the same vision; for instance, Amazon is loading ChatGPT into its Alexa devices and Boston Dynamics is enabling their robots to speak after the integration with ChatGPT. Finally, as a selected example, a recent study conducted by Tafferner et al [26] presented interesting attempts toward the integration of ChatGPT with temperature, light, motion, humidity, gas, water, or smoke sensors in the smart home setting, and ChatGPT was found to be able to even facilitate the design of such smart home systems and thus reduce the workload of not only health care professionals but also system engineers.

## Development of Personalized or Precise Digital Health Interventions

It is not surprising that nowadays people are asking ChatGPT various medical and health care questions, including available therapies for specific diseases or options for symptom management. As a commercial product made available to the public, ChatGPT is expected to give similar answers to the same question even if asked by different people. However, a natural question to ask is whether LLMs can learn individual user preferences like many other AI tools do, such that ChatGPT can adapt to a specific user’s trajectory (eg, inquiry history, content of interest, satisfaction with answers, and type and frequency of purposeful use) and thus may provide customized contents in a personalized tone. This is a question of significant interest to both users and researchers. A very recent article by the Brain Team of Google Research [27] just tackled this exact question by evaluating the effectiveness of an LLM-based recommendation system, and their results suggested the feasibility and potential of such concepts. An alternative solution is to create a personal version of ChatGPT (also known as private and individualized AI), the training and operation of which should be afforded through a PC rather than high-performance computing clusters. There are multiple ongoing efforts in both directions, and the good news is that tools such as MiniGPT-4 have been made available to the public and they are intended for individual use. Personalized ChatGPT could substantially renovate the ecosphere of digital health. Like oncology treatment, many one-size-fits-all digital health therapies or interventions have been proven less effective or futile in terms of minimizing harms and maximizing benefits to an individual. There is a need for precision oncology treatment in a similar way for precision digital health therapies or interventions. Using nonpharmacological neuromodulation (eg, transcranial magnetic or electrical stimulation) as an example, it remains unclear to researchers what factors or mechanisms dictate whether and how an individual will positively respond to open-loop neuromodulation. This is an important question as the percentage of nonresponders found in previous digital health studies could be 20% or higher. Personalized ChatGPT is expected to help our researchers to better understand individual-level behavioral patterns, stress levels, vital sign trajectories, dietary habits, health care needs, and even instant treatment responses. We also expect a gradual shift of classical clinical trial design paradigms toward an AI-assisted N-of-1 trial design in digital health. Toward such goals, the recent work of Singhal et al [7] should be noted as a representative example since they introduced an expert-level medical question–answering platform based on LLM, which can be tailored to specific health care domains and eventually be personalized as an individual’s health care assistant after appropriate data feeding and parameter tuning.

## Improve Analytics to Enable Easier Interpretation and Better Clinical Decision-Making

NLP research and its applications have a long history that can be traced back to Turing’s thinking machine and the Hodgkin-Huxley model in the 1950s. However, analytic power had never been the focus of NLP methodology development before ChatGPT was launched. It turned out to be a big surprise to many users that ChatGPT, as an NLP product, can carry out computer coding and data analytics even better than many entry- to moderate-level professionals. While it is well known that more than 145 million dialogues extracted from various sources (eg, Twitter, Reddit, and Wikipedia) have been used to train ChatGPT, people may wonder what, in addition to the power of big data, suddenly grants LLMs analytical skills. We hypothesize that such observation should be attributed to the power of language itself. Specifically, language data are different from many other data types in the sense that instead of cold numbers, language data contain rich amounts of human thinking, logic, knowledge, behavioral patterns, etc. Anyway, language data are a type of so-called sequence data. Since LLMs are capable of processing and understanding language data well, we speculate the possibility of using LLMs for other types of sequence data such as multivariate time series. In the digital health field, a big volume of various sequence data, including but not limited to vital sign signals, electronic health or medical records, image streams, clinical genetic or metabolic profiles, and ambient sensor signals, are being generated every day from, for example, hospitals, clinics, outpatient organizations, communities, and individuals worldwide. Such big and complex sequence data present serious challenges to the analytic capabilities of many existing algorithms and tools. LLMs such as ChatGPT could be an alternative framework to better address digital health data analytical problems. For recent progress along this line of ideas, BERT-log [28] and AnomalyBERT [29] for anomaly detection in sequence data can be noted. Also, it should be noted that OpenAI recently used GPT-4 to explain every neuron in GPT-2, which suggests a different but promising direction of using ChatGPT’s analytic power for deep learning interpretation. Further examples of ChatGPT’s applications in health care analytics and decision-making include the work of Hirosawa et al [30] for differential diagnosis list generation and that of Rao et al [31,32] for clinical decision-making support including cancer screening.

## Privacy and Ethical Issues of Trustworthy GPT Tools for Digital Health

With the right training data and right model structure, ChatGPT is now able to speak like a human; however, it has not evolved sufficiently to think like a human, which is a double-edged sword for humanity. Even at the current stage of its maturity, numerous concerns regarding privacy, ethics, job security, education policies, etc, have been raised regarding the use of ChatGPT in various scenarios. In digital health, the story is the same. The most common way of using ChatGPT is to input questions or upload digital documents (or both). It is possible that such information is intercepted by malicious third parties, causing serious privacy concerns especially when protected health information is involved. Currently, it is unclear to the public how OpenAI will detect and protect such sensitive information. Another common concern for digital health professional communities is the possibility of a scientist either writing or reviewing manuscripts or grant applications using ChatGPT. Given the many years of training and experience of a qualified scientist, using ChatGPT to carry out such work looks “reckless” and “not scientific” to many of our peers. The other side of the story is that researchers constantly get assistance from various colleagues, mentors, training courses, and scientific writing specialists. Therefore, using ChatGPT as an assistant tool may resemble the aforementioned kind of help and may thus be acceptable. However, the real situation is trickier than that. One must consider the possibility of a scientist asking ChatGPT certain questions about a manuscript’s central idea that is new to the professional community or about the novel specific aims of a grant proposal, or generating fake information or data (or both). When someone feeds such questions or information into ChatGPT, they become a part of its knowledge base; this will make confidential or misleading information available to the whole system and to general users. The development of safeguards that can protect privacy and avoid ethical concerns when using GPT tools should be on our digital health scientific communities’ agenda. Technically, machine “unlearning” [33] might be one of the potential solutions for GPT tools to “forget” certain information and thus protect privacy and reduce ethical concerns.

Despite major debates regarding the use of GPT tools for education, research, and practice purposes, here we have presented a perspective and pioneering examples in the digital health field where GPT tools have the potential to enhance and empower a smarter future of digital health or more intelligent ways to optimize digital health applications in reducing clinician burden, simplifying communications and workflow in health care, and personalizing digital health interventions for precision treatment responses. In short, considering various risks and concerns of using GPT in digital health, we would like to call on all GPT developers, digital health researchers, health care practitioners, and policy makers to coevolve smarter digital health technologies while developing privacy and ethical rules and policies embedding humanity principles in this transformative GPT era in health and health care.

## Abbreviations

AI: artificial intelligence
GPT: Generative Pretrained Transformer
LLM: large language model
NLP: natural language processing
